# Downtime after Critical Incidents in Emergency Medical Technicians/Paramedics

**DOI:** 10.1155/2014/483140

**Published:** 2014-05-04

**Authors:** Janice Halpern, Robert G. Maunder, Brian Schwartz, Maria Gurevich

**Affiliations:** ^1^Department of Pychiatry, Mt. Sinai Hospital and the University of Toronto, Toronto, Canada M5G 1X5; ^2^Public Health Ontario and Departments of Family and Community Medicine and Dalla Lana School of Public Health, University of Toronto, Toronto, Canada M5G 1V2; ^3^Sunnybrook Osler Centre for Prehospital Care, Toronto, Canada M4N 3M5; ^4^Department of Psychology, Ryerson University, Toronto, Canada M5B 1X5

## Abstract

Effective workplace-based interventions after critical incidents (CIs) are needed for emergency medical technicians (EMT)/paramedics. The evidence for a period out of service post-CI (downtime) is sparse; however it may prevent posttraumatic stress disorder (PTSD) and burnout symptoms. We examined the hypothesis that downtime post-CI is associated with fewer symptoms of four long-term emotional sequelae in EMT/paramedics: depression, PTSD, burnout, and stress-related emotional symptoms (accepted cut-offs defined high scores). Two hundred and one paramedics completed questionnaires concerning an index CI including downtime experience, acute distress, and current emotional symptoms. Nearly 75% received downtime; 59% found it helpful; 84% spent it with peers. Downtime was associated only with lower depression symptoms, not with other outcomes. The optimal period for downtime was between <30 minutes and end of shift, with >1 day being less effective. Planned testing of mediation of the association between downtime and depression by either calming acute post-CI distress or feeling helped by others was not performed because post-CI distress was not associated with downtime and perceived helpfulness was not associated with depression. These results suggest that outcomes of CIs follow different pathways and may require different interventions. A brief downtime is a relatively simple and effective strategy in preventing later depression symptoms.

## 1. Introduction

Emergency medical technicians (EMT)/paramedics experience considerable workplace stress. This is reflected in various recent comparisons of physical and emotional health measures. For instance, in a comparison with 25 other occupations, EMT/paramedics scored highest in physical symptoms, second highest in job dissatisfaction, and fourth highest in psychological difficulties [1]. The burden of stress for this occupational group is thought to be related to critical incidents, events that arouse intense distress which may interfere immediately with functioning or result in later emotional sequelae [2]. Critical incidents often involve patient death [3] or a feeling of inability to help on the part of the EMT/paramedic [4]. It is not surprising, then, that the UK National Health Service annual sickness-absence rates are repeatedly the highest in ambulance workers [5]. EMS organizations have a responsibility to prepare their employees for critical incidents and to provide postincident strategies to mitigate their effects [6]. Over the past two decades, the efficacy of critical incident stress debriefing (CISD), the most relied-upon postincident mitigating strategy, has come increasingly into question [7, 8]. CISD is usually conducted in groups within a few days of the incident. It includes sharing of thoughts and feelings about the incident, as well as psychoeducation. Recent randomized control trials have failed to show a unique effect of CISD on posttraumatic symptoms, anxiety, or depression [9, 10] in high-risk occupations. This leaves EMS organizations without evidence-based postcritical incident interventions to offer their employees. The effectiveness of other potential strategies needs to be studied.

Most EMT/paramedics expect that intervention after a critical incident will be provided in the workplace and many prefer this location [11, 12]. The workplace setting likely helps them maintain or regain control and confidence in their abilities [11]. However, there are no empirical studies to our knowledge on the effectiveness of interventions in the workplace to improve the short-term and long-term emotional outcomes of critical incidents. We chose to explore the value of providing downtime for EMT/paramedics soon after a critical incident, a strategy that some EMS organizations employ. We define the term “downtime” as a period of being out of service after a critical incident. Usually downtime is granted by management when an EMT/paramedic reports a critical incident and some indication of related distress. At other times downtime is naturally available, such as when a critical incident occurs just before a break or end of a shift. Organizations and individual supervisors vary in their willingness to offer downtime, and EMT/paramedics also vary in their willingness to request it. Barriers to downtime have been studied [13] and include the time pressures that are inherent in EMS organizations and a culture that stigmatizes vulnerable emotions. Barriers to supervisors granting downtime include difficulty in recognizing and feeling comfortable with emotions, as well as a conviction that vulnerable emotions are inappropriate in the workplace. EMT/paramedic barriers include fear of stigma, expecting an unsupportive response, not recognizing the incident as critical, or avoiding thinking or speaking about the incident [13].

Practically speaking, downtime would be a fairly simple intervention to adopt for organizations that have not done so already. Unlike CISD, downtime only targets distressed individuals and requires no outside professionals. It does, however, entail the cost of taking affected EMS/paramedics out of service for some period of time. Optimally, downtime would also entail educating EMS/paramedics and supervisors to report and respect expressions of distress and to value this intervention.

A few studies of downtime have been published. Two studies of first responder groups have identified deleterious effects of insufficient time to recover from critical incidents. In their study of police officers, Carlier et al. [14] found that insufficient time for "coming to terms" with a traumatic incident predicted PTSD symptoms 3 months later, although there was no longer an effect after 12 months. This led the authors to suggest that police organizations allow their employees “some time for rest” before returning to work. A survey of ambulance workers [15] revealed an association between the response of “never” to the question of how often they had time to recover between incidents and high emotional exhaustion scores on the Maslach Burnout Inventory. This survey also found that over two-thirds of subjects reported insufficient time to recover between events.

In Ørner's survey, emergency services personnel endorsed a “wait and see” attitude after critical incidents, with an emphasis on rest, relaxation, and reestablishing control. Talking about the incident was also strongly endorsed but in a context of EMT/paramedics' own choice [11]. In a more recent qualitative study, ambulance workers described the experience of a postincident downtime, which they clearly valued [13]. They described a brief period of (1/2)–1 hour duration, during which the worker is taken out of service by his or her supervisor. The time was usually spent informally with peers who often had participated in the same incident, and sometimes their supervisor would join them. The conversation ranged over a variety of topics, including the incident. Some preferred to mostly listen. They described it as a time to relax, “decompress,” or “vent” with trusted individuals by whom they felt understood. The use of downtime in response to patient death has been touched upon in two other health care groups. After an inquiry had recommended that surgeons refrain from operating for 24 hours after an intraoperative death, a survey of orthopaedic surgeons found that 15 of 16 surgeons who experienced an intraoperative death continued to operate that day [16]. A similar survey of 250 anesthesiologists found that a majority considered this a reasonable recommendation, but only one-quarter of respondents thought this was practical. They suggested that provisions be made for those who might feel the need to take this time [17].

Our primary goal in this study was to examine the relationship between a downtime period after a critical incident and long-term emotional sequelae. We chose symptoms of four emotional sequelae which appear in the literature on critical incident stress in first responder groups: depression [18], posttraumatic stress [18], burnout [15], and stress-related physical symptoms [19]. A review of studies on paramedic health [20] stated that the prevalence of posttraumatic stress disorder (PTSD) in paramedics is 12% to about 20%, compared to reported community prevalence of 1–3%. Since PTSD is clearly tied to acutely stressful incidents, the high rates in EMT/paramedics are likely due to their greater exposure to such incidents. Although the review's authors found no comparable studies in the community on depression, they reported the prevalence in EMT/paramedics as also about 20%. Both PTSD and depression have been shown to occur after traumatic stress [21], although chronic workplace stress or other factors may contribute to both. Critical incidents have been implicated in the development of burnout [15]. Stress-related physical symptoms have been connected with the “psychological demands” of the job [19].

Our secondary goal was to explore possible mediators of any relationship found between downtime and later emotional sequelae. One hypothesis was that, since downtime is usually taken soon after the incident, its initial effect would be to decrease the acute anxiety aroused by a critical incident. Since there is evidence that faster recovery from the acute stress of a CI is associated with fewer later emotional sequelae [12], we expected that this early calming might mediate the relationship between downtime and long-term emotional sequelae. Rapid recovery from acute stress is also important in itself, since there is evidence that acute stress affects EMT/paramedics' work performance [22]. Acute stress can be measured by considering the components of the acute stress reaction (ASR) [23], which is a response to extreme stress that usually lasts for up to 2 or 3 days. Some of the common components of the ASR that are easily identified by paramedics are insomnia, physical arousal sensations (palpitations, sweating, and shaking), irritability, social withdrawal, and distressing feelings [24].

We also hypothesized that a second potential mediator of the effect of downtime on later sequelae might be the feeling of social support engendered by a feeling of being helped by others. This help could refer either to the provision of downtime by the organization or interactions with others during downtime. Social support has long been identified as protective against PTSD in high-risk occupations [25].

## 2. Materials and Methods

This study was part of a larger study on risk and resilience in ambulance workers, specifically EMT and paramedics. Ambulance workers, both front-line and supervisors, were recruited from attendees of a mandatory continuing medical education programme (CME) in a large urban EMS organization. A recruitment letter informed ambulance workers on leave of absence about the study since they did not attend the CME programme. Subjects completed their choice of paper or web-based survey. Participants' names were entered into a draw for monthly prizes worth up to $600. University research ethics board approval was obtained.

The survey enquired about two time periods. There were questions about the acute stress following the index critical incident chosen by each subject. These covered the period from the time of the incident until a few months later. Questions about the long-term sequelae refer to the time at which the survey was being completed by the subject.

### 2.1. Instruments

#### 2.1.1. Demographics

This included age, gender, marital status, years of service, level, or job title.

#### 2.1.2. Critical Incidents

We asked participants how many critical incidents they had experienced. We defined critical incidents as “calls that have generated unusually strong feelings, either because of the incident itself, or how it was handled or some other reason”. In order to maximize the opportunity to identify an index incident, participants were asked first to identify an incident that was “still troubling.” Those who could not identify a still troubling incident were asked to identify an incident that “had been troubling in the past.” Failing this, they were asked to describe “a composite of a number of critical incidents.” Finally, those who were unable to describe a composite were asked to describe “one of your worst calls.” We also enquired about how long ago the index incident took place or over what time period in the case of the composite.

#### 2.1.3. Duration of Acute Stress Reactions to Critical Incident

We measured five components of the acute stress reaction in response to the index critical incident. The components measured were physical arousal reactions “like sweating, shaking and pounding heart,” distressing feelings “like fear, anger, horror, guilt, shame worry or sadness,” disturbed sleep “sleep disrupted by the incident,” irritability “irritable, mean or snappish,” and social withdrawal “if you withdrew or pulled back from other people.” For each dimension, participants reported the occurrence in response to the incident and how long it took to get back to normal by choosing one of seven options: (i) did not have this reaction or returned to normal (ii) soon after the call (a few hours), (iii) by the next night, (iv) by the next week, (v) by the next month, (vi) within a few months, or (vii) still not normal. Based on the results of a previous study of acute stress symptoms in this cohort [12], distressing feelings, insomnia, irritability, and social withdrawal were dichotomized as persistent (>one night) or not, and physical arousal was dichotomized as any/none. However, because downtime could not plausibly affect physical arousal until after the CI, physical arousal was also analyzed as physical arousal persisting beyond one night (yes/no).

#### 2.1.4. Downtime

Due to the paucity of literature on downtime, survey questions were constructed to have face validity. Participants were asked “How much time did you have to try to deal with your feelings after the situation?,” with choices of “no time,” “less than 30 minutes,” “30 minutes to 2 hours,” “rest of the shift,” and “a day or more.” Unless a respondent reported having had “no time,” we then asked how much of this downtime was “paid time off given by your supervisor,” and we dichotomized responses as any paid downtime versus no paid downtime. Participants were asked how helpful the time was “in getting hold of your thoughts and feelings,” and offered 5 choices from “very unhelpful” to “very helpful.” Responses were collapsed into three categories: helpful (including helpful and very helpful), neutral, and unhelpful (including unhelpful and very unhelpful). Participants then chose from a list all the persons they had spent time with during downtime or available time. This list included various members of the organization, as well as family and friends.

#### 2.1.5. Psychological Symptoms at Time of the Survey

*Center for Epidemiologic Studies Depression Scale, Short Form (CES-D-10)*. This 10-item scale is the short version of the CES-D. Responses rated the frequency of depressive phenomena on a 4 point scale from 0 (rarely or none of the time, less than one day) to 3 (all of the time, 5–7 days). The scale is scored as the sum of all item scores. CES-D-10 scores show concurrent validity with measures of positive affect (*r* = −63) and poor health status (*r* = 37). The 10-item scale is highly correlated with the CES-D, which has been validated against clinical diagnoses of depression [26]. The cut-off score for the CES-D has been validated with DSM-III major depression. The CESD-10 cut-off score of 10 discriminates consistently with the cut-off score for the original [27]. Internal reliability was 0.77.

*Impact of Events Scale-Revised*. This 22-item measure of traumatic stress probes the intensity of responses to a particular event on a 5-point scale from 0 (not at all) to 4 (extremely). The scale is scored as the mean of item scores. The IES-R yields 3 subscales (avoidance, intrusion, and hyperarousal) and a total score. The three subscales have strong internal consistency and satisfactory test-retest reliability [28]. The correlation between the Mississippi Scale for Combat-Related PTSD, Civilian Version, and the three subscales of the IES-R were intrusion, *r* = 53, avoidance, *r* = 55, and hyperarousal, *r* = 55 [29]. A cut-off of ≥1.5 has been used to identify possible cases. Internal reliability for total scale was 0.91.

*Brief Symptom Inventory (BSI) Somatization Subscale (Measure of Stress-Related Physical Symptoms)*. The BSI is abbreviated from the Symptom Checklist 90-Revised. The 7-item somatization scale probes how much the participant was distressed by the discomfort of a physical symptom using a 5 point scale, from 0 “not at all” to 3 “extremely.” The SCL-90 has demonstrated reliability and validity [30]. The BSI-somatization scale has been validated against the SCL-90R and comparable scales of the MMPI. To identify cases, a cut-off was set at the value of the mean + 1 standard deviation in a nonpsychiatric patient normative sample (cut-off = 0.69) [31]. Internal reliability was 0.79.

The time period for the three scales above was altered from the standard “over the last week” to “your current or most recent block of shifts on duty” because in a pilot study ambulance workers reported that psychological distress was worse during blocks of shifts on than during time off.

*Maslach Burnout Inventory Human Services Survey-Emotional Exhaustion Scale*. This questionnaire inquires about present job-related feelings. Responses describe the frequency of phenomena in seven categories from 1 (never) to 7 (every day). There is strong psychometric evidence of both reliability and validity for three subscales. A cut-off of 27 on the 9-item emotional exhaustion subscale was used to identify burnout, based on the recommendations of the scale's authors [32].

### 2.2. Statistical Analysis

Differences between means were tested by ANOVA. Differences in the prevalence of categorical variables were tested by Pearson chi-square test. Mediation analysis according to the method of Baron and Kenny [33] was planned. Significance was set at *P* < 05 (two-sided). Statistical tests were conducted using IBM SPSS (version 22).

## 3. Results

Nine hundred and six ambulance workers were informed of the study, 635 who provided consent received the survey, and 243 (38.3%) returned it. Of these there were 217 valid responses for the questions on downtime, short-term (acute stress reaction), and long-term emotional outcomes. Of these 217, 201 completed an instrument measuring at least one of burnout (*n* = 192), depression (*n* = 196), posttraumatic stress symptoms (*n* = 187), or stress-related physical symptoms (*n* = 199) and were included in this analysis.

Of these 201 participants, 127 (63%) were men and 73 (37%) women (1 not answered). Mean age was 37.6 (standard deviation, SD: 9.4 and range: 22–59). Level of training (beginning with basic) was distributed as 84 (42%) level 1 (EMT), 38 (19%) level 2 (intermediate - EMT with some paramedic skills), 71 (35%) level 3 (paramedic), and 4 (2%) supervisors (4 not answered). Mean years of service were 7.6 (SD: 3.3 and range: 1–12). Sixty-four (32%) were single, 123 (61%) were married or common-law, and 13 (7%) were divorced or separated (1 not answered). These demographics were representative of the organization, except female gender and the highest level of training were overrepresented in the study sample. In the organization, 24% of all ambulance workers were female and 25% had level 3 training at the time of the study.

The characteristics of the index critical incident were as follows. One hundred and five participants (52%) reported on an incident that was still troubling, 76 (38%) reported on an incident that was troubling in the past, 4 reported on a composite because a single incident was difficult to isolate, and 14 (7%) reported on their “worst call” (indicating that they did not endorse having experienced a critical incident). The index critical incident had occurred within the last year for 48 participants (27%), while 79 (45%) were experienced within 5 years of the study, and for 50 (28%) more than 5 years had elapsed. Fifty-four participants (27%) received no downtime for the index critical incident. Thirty-six (18%) received less than 30 minutes; 54 (27%) received 30 to 120 minutes; 24 (12%) received the remainder of the shift as downtime; and 33 (16%) reported a day or more of downtime after the CI.

Of the 147 paramedics who received downtime, some portion of the downtime was paid for 70 (48%), none was paid for 73 (50%), and 2 (1%) did not report if downtime was paid or unpaid. Most commonly, paramedics reported spending downtime with another paramedic who was at the scene (93, 63%), another paramedic not at the scene (31, 21%), a supervisor (32, 22%), family (41, 28%), and/or a friend (21, 14%). Nineteen (13%) found the downtime very unhelpful or unhelpful, 34 (23%) found it to be neutral, and 87 (59%) found it to be helpful or very helpful (not reported by 7, 5%).

Receiving any downtime was associated with significantly lower depressive symptoms (any downtime: mean 6.9 ± SD 4.3 and none: 8.9 ± 5.1; *P* = .008). Downtime was not significantly associated with posttraumatic symptoms (any: 0.71 ± 0.59 and none: 0.78 ± 0.61; *P* = .48), burnout (any: 21.2 ± 11.1 and none: 24.1 ± 12.3; *P* = .12) or somatic symptoms (any: 0.43 ± 0.40 and none: 0.55 ± 0.46; *P* = .07). Therefore, posttraumatic stress symptoms, burnout, and stress-related physical symptoms were excluded from subsequent analyses.

Mean depressive symptoms score declined significantly with the increasing duration of downtime on the day of the critical incident (no time: 8.9 ± 5.1; <30 min: 7.3 ± 4.2; 30 min–2 hr: 6.6 ± 4.3; rest of shift: 6.0 ± 3.6; df = 3; *F* = 3.3; *P* = .02). However, time-out lasting > 1 day was associated with somewhat higher mean scores (7.6 ± 4.6).

Table 1 indicates that particular aspects of downtime, specifically whether it was paid or unpaid and whether or not it was perceived as helpful, were not significantly associated with depressive symptoms.

Receiving any downtime was not significantly associated with faster recovery from any of the symptoms of acute stress, namely, insomnia (any downtime: 43%, no downtime: 56%, *χ* ^2^ = 2.7, and  *P* = .12), irritability (any downtime: 29%, no downtime: 33%, *χ* ^2^ = 0.3, and *P* = .61), social withdrawal (any downtime: 30%, no downtime: 32%, *χ* ^2^ = 0.1, and *P* = .73), distressing feelings (any downtime: 55%, no downtime: 53%, *χ* ^2^ = 0.1, and *P* = .87), or physical arousal (any downtime: 7%, no downtime: 13%, *χ* ^2^ = 1.6, and *P* = .26). As a result the planned mediation analysis was not performed.

The perception of helpfulness of downtime was not associated with depressive symptoms, which also disqualified it as a mediator of the relationship between receiving downtime and depressive symptoms.

## 4. Discussion

In summary, nearly 75% of the participants received downtime, almost evenly divided between paid time and time that happened to be available (e.g., end of shift). The vast majority (84%) spent the time with peers, and most (59%) found it helpful or very helpful. Longer downtime was associated with lower depression scores, up to and including a full day. Longer periods were not associated with lower depressive scores. Recovery from acute stress was not associated with receiving downtime, and perceived helpfulness of downtime was not associated with depressive symptoms, such that both of these were disqualified as potential mediators of the association between downtime and lower depression scores. Symptoms of posttraumatic stress, burnout, and stress-related physical symptoms were not significantly associated with receiving downtime.

To our knowledge, this is the first quantitative study to explore and show a relationship between depressive symptoms and downtime in EMT/paramedics or any other first responder group (firefighters and police officers). In Carlier et al.'s study, at one year postincident there was also no effect of downtime on PTSD symptoms, although there was an effect at three months postincident [14]. Based on a previous study of the same cohort [12], high depressive symptom scores were present in 24%, while posttraumatic stress scores were only present in 8%. This suggests that depression symptoms are a significant concern in this population.

Our finding that downtime is not associated with early calming of the acute stress reaction and yet is associated with lower depression scores suggests that early calming is not the mechanism by which downtime is associated with depression. Early calming has been shown to be associated with fewer PTSD symptoms, but its connection with fewer depression symptoms is not as strong [12, 34–36]. Thus there seems to be a characteristic of downtime that is not calming and does not mitigate PTSD, but does mitigate depression. Shalev et al.'s 1998 study on depression and PTSD following trauma tellingly noted that “early autonomic activation may be specifically linked with subsequent PTSD, while the mechanisms that mediate the occurrence of depression may be of a different nature” [21]. Another example of a possible difference between the development of PTSD and depression is the lack of association between the perception of helpfulness of downtime (feeling socially supported) and depressive symptoms in this study. In contrast, an association has been found elsewhere between social support and PTSD [25]. The mediators of the effect of downtime on depression after critical incidents clearly require further investigation. One hypothesis is that a time to reflect on the incident on one's own terms increases feelings of control and self-efficacy, which are important in maintaining feelings of competence and, in doing so, averting depressive symptoms. Thompson and Suzuki [37] noted that the coping strategies used by ambulance workers reflected strategies used when self-esteem is threatened and Halpern et al. [4] note the importance of feelings of helplessness and incompetence in the face of the critical incident in predicting outcomes. Beginning to regain pride in one's work and come to grips with the impossibility of perfection may be part of what happens during downtime, especially in the presence of peers and understanding supervisors. This is different from calming and the safety of relying on others, which may be more important in posttraumatic stress than depression. Another possibility for mediation between downtime and depression is the temporary decrease in the time pressure of this very time-sensitive and demanding job and delay of further exposure to potentially stressful incidents which allows for the beginning of recovery. This may mean allowing oneself to explore internally one`s feelings and thoughts about the incident, which is helpful to recover [38]. Thus, how an individual uses downtime—whether to avoid feelings or begin to process them—may colour its effectiveness.

The optimal length of time for downtime appears to be less than one day. This may be because a prolonged time off work encourages avoidance coping strategies, which prevent processing of the incident [39]; alternatively it may be that downtime for more than a day is a marker of greater psychological burden and thus of risk for worse outcomes. While this is known to interfere with processing of posttraumatic symptoms, it may also be the case for depressive symptoms [38].

Since the questions on the index critical incident and the early stress reactions rely on memory for incidents as recent as less than six months to as long as over 5 years, this study is limited by recall bias. The lag time between the index critical incident and the measurement of outcomes also allows further traumas to have occurred, both within and beyond the workplace. The term “cumulative trauma” [40] refers to the risk of repeated traumatization in high-risk professions and makes us mindful that the outcomes often cannot be traced to a single incident. This study is also limited by the cross-sectional method, which allows comments on association rather than causality. The participation rate is relatively low, which is likely due to a number of factors. Because of concerns about confidentiality, the questionnaires were not completed in the workplace, such that these busy professionals were required to spend their scarce free time completing them. Although we attempted to keep our questions to the minimum, future studies may want to reduce the burden of participation by further streamlining the questionnaires to increase response. In addition the most distressed individuals may have avoided participation out of concerns that it might exacerbate their symptoms, and the least distressed may have had little interest in participating in the study. Another limitation is the self-selection of the subjects, although the demographics of the participants are fairly representative of the organization. Finally, since the study was limited to one urban EMS service, results may not be generalizable to all EMT/paramedics.

## 5. Conclusions

Downtime after a critical incident is significantly associated with lower depressive symptoms scores in EMT/paramedics on long-term follow-up. This association is mediated by neither faster recovery from acute stress nor feeling helped by others during the downtime. The optimum length of downtime seems to be up to one day. Since depression is an important long-term outcome of critical incidents in EMT/paramedics, a brief downtime period may be a worthwhile intervention for EMS organizations to adopt. Future studies could include a prospective design and measures of cost-effectiveness of this workplace intervention.

## Figures and Tables

**Table 1:** Aspects of downtime and depressive symptoms.

	*N*	Depressive symptoms	SD	df	*F*	*P*
		Mean				
No downtime	51	8.9	5.1			
Any downtime	145	6.9	4.3	1	7.3	.008
Qualities of downtime						
Paid	70	7.0	4.3			
Unpaid	70	6.7	4.1	1	0.18	.68

Perceived as unhelpful	19	6.7	4.3			
Perceived as neutral	34	7.3	4.6			
Perceived as helpful	86	6.5	4.1	2	0.4	.66
